# Nutrient Sensing: Another Chemosensitivity of the Olfactory System

**DOI:** 10.3389/fphys.2017.00468

**Published:** 2017-07-12

**Authors:** A-Karyn Julliard, Dolly Al Koborssy, Debra A. Fadool, Brigitte Palouzier-Paulignan

**Affiliations:** ^1^Univ Lyon, Université Claude Bernard Lyon1, Centre de Recherche en Neurosciences de Lyon (CRNL), INSERM U1028/Centre National de la Recherche Scientifique UMR5292 Team Olfaction: From Coding to Memory Lyon, France; ^2^Department of Biological Science, Florida State University Tallahassee, FL, United States; ^3^Program in Neuroscience, Florida State University Tallahassee, FL, United States; ^4^Institute of Molecular Biophysics, Florida State University Tallahassee, FL, United States

**Keywords:** nutrient sensing, olfaction, piriform cortex, transporter, receptor, food intake, obesity, type 2 diabetes

## Abstract

Olfaction is a major sensory modality involved in real time perception of the chemical composition of the external environment. Olfaction favors anticipation and rapid adaptation of behavioral responses necessary for animal survival. Furthermore, recent studies have demonstrated that there is a direct action of metabolic peptides on the olfactory network. Orexigenic peptides such as ghrelin and orexin increase olfactory sensitivity, which in turn, is decreased by anorexigenic hormones such as insulin and leptin. In addition to peptides, nutrients can play a key role on neuronal activity. Very little is known about nutrient sensing in olfactory areas. Nutrients, such as carbohydrates, amino acids, and lipids, could play a key role in modulating olfactory sensitivity to adjust feeding behavior according to metabolic need. Here we summarize recent findings on nutrient-sensing neurons in olfactory areas and delineate the limits of our knowledge on this topic. The present review opens new lines of investigations on the relationship between olfaction and food intake, which could contribute to determining the etiology of metabolic disorders.

## The olfactory system is an interface

According to its anatomical location, the olfactory system is well poised to be an interface, with the ability to gather and process information simultaneously from the external and internal environment.

### Interaction with the external environment

The traditional function of the olfactory system is to sense the external chemical world. Odors are inhaled directly into the nose following an orthonasal pathway, or come from the back part of the mouth following a retronasal pathway. Both pathways lead odors to the posterior part of the nasal cavity. Odors bind to protein receptors located in the ciliary membrane of olfactory sensory neurons (OSNs) within the olfactory epithelium (OE). Each OSN expresses only one type of olfactory receptor (Malnic et al., 1999; Serizawa et al., 2003). Odor/receptor association selectively activates OSNs in the OE. All OSNs expressing the same odorant receptor project their axons to one or two olfactory bulb (OB) glomeruli where OSN axons synapse with the dendrites of mitral cells (MCs); the second order olfactory neurons (Ressler et al., 1994; Vassar et al., 1994; Breer et al., 2006). The electrical signal is then transmitted to neuronal networks in the piriform cortex (PC). Olfaction thereby informs the central nervous system in real time about the chemical composition of the external environment prior to any visual or tactile information. This event allows the animal to anticipate and rapidly adapt its behavior when seeking food or when engaging in social or sexual behavior.

### Interaction with the internal environment

**Figure 1:**
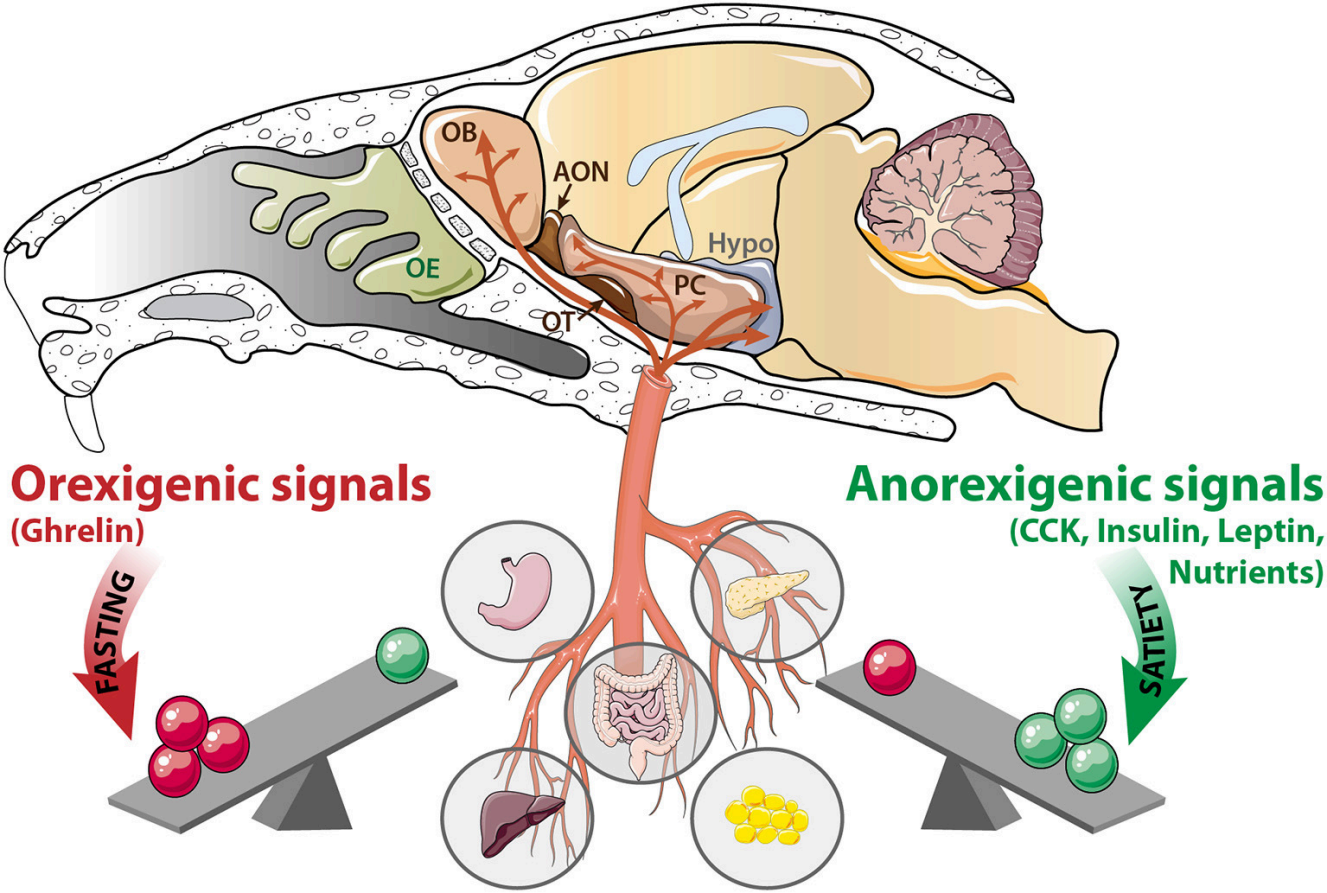
The olfactory system is a metabolic sensor like the hypothalamus. According to the nutritional status, a balance exists between peripheral signals delivered by the stomach, intestine, liver, pancreas, and adipose tissue. During fasting, orexigenic signals (ghrelin, and nutrients scarcity) prevail. In contrast, during satiation, anorexigenic signals (CCK, insulin, leptin and nutrients abundance) are predominant. These signaling molecules reach the central nervous system via the blood flow, where they target the hypothalamus (Hypo) as well as a variety of olfactory structures: OE, olfactory epithelium; OB, olfactory bulb; AON, anterior olfactory nucleus, OT, olfactory tubercle; PC: piriform cortex; CCK, cholecystokinin.

The hypothalamus is the main central actor in food intake regulation. Internal signals carried by the blood inform various central areas about the body's fuel availability, which in turn implement appropriate behavioral and metabolic responses to physiological requirements. Orexigenic and anorexigenic signals, respectively, stimulate or inhibit food intake by modulating neuronal activity of hypothalamic nuclei. During fasting, the hypothalamus induces food intake in response to nutrient scarcity and high level of ghrelin released by the stomach. Alternatively, the hypothalamus suppresses feeding behavior when it detects insulin secretion from the pancreas, leptin secretion from the adipose tissue, and nutrient abundance (Blouet and Schwartz, 2010; Berthoud, 2011). Interestingly, the olfactory system is also becoming widely considered as an active sensor of internal signaling (hormones, micronutrients availability). Olfactory structures like the OE, OB, and PC (Palouzier-Paulignan et al., 2012) express high levels of various hormone receptors (insulin, leptin, ghrelin, CCK) similar to that of the hypothalamus (Figure 1). When targeting their receptors, metabolic hormones modulate the electrical activity of olfactory networks (Fadool et al., 2000, 2011; Apelbaum et al., 2005; Hardy et al., 2005; Lacroix et al., 2008; Savigner et al., 2009; Kuczewski et al., 2014). OB neurons respond not only to peptides, but they also respond to glucose and express molecular hallmarks of glucose sensing cells (Tucker et al., 2010, 2013; Aimé et al., 2014; Al Koborssy et al., 2014; Kovach et al., 2016).

The metabolic sensing function of the OB is consistent with its high density of capillary network (Chaigneau et al., 2007) and its vascular properties. The blood brain barrier of the OB is not as tight as it is in the cerebral cortex or other brain regions (Ueno et al., 1991, 1996), indicating that blood-borne metabolic signals can enter the OB more easily than other brain regions. The permeable blood barrier facilitates transport of intravascular macromolecules, including nutrients and peripheral hormones, and their direct action on the OB. This enhanced permeability allows adaptation of olfactory perception to the physiological state: highly sensitive when the animal is fasted and needs to find food, and slightly sensitive when the animal is satiated (Aimé et al., 2007, 2012; Julliard et al., 2007; Prud'homme et al., 2009; Tong et al., 2011). Based upon its sensitivity to metabolic hormones and glucose availability, the olfactory system is proposed to be a metabolic sensor.

The present review provides an updated outlook of nutrient sensing in olfactory structures. We argue that in addition to being glucose-sensitive (Tucker et al., 2010, 2013; Aimé et al., 2014; Al Koborssy et al., 2014; Kovach et al., 2016) olfactory structures are sensors of amino acids (AAs) and potentially of fatty acid (FA) content of the internal medium.

## Transmembrane protein families involved in nutrient sensing

**Figure 2:**
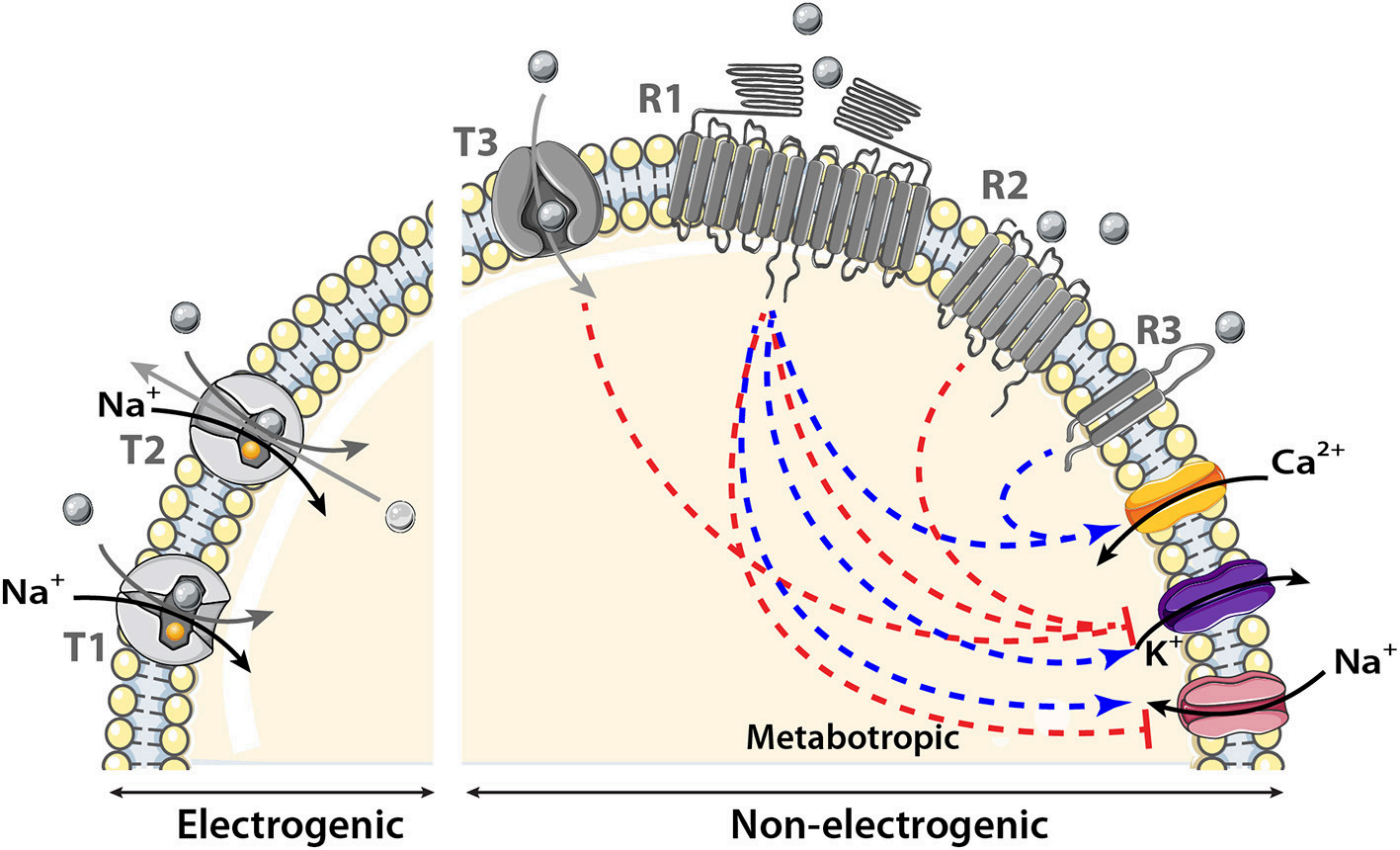
Schematic model showing the transmembrane proteins involved in nutrient sensing. The main transporter (T) family involved in nutrient sensing is the solute carrier (SLC) transporter family. It couples the movement of the nutrient (gray circle) to that of another molecule or ion crossing the membrane either in the same (symporter) named T1 in the figure or opposite direction (antiporter or exchanger) named T2 in the model. Nutrient influx down SLC transporters is called electrogenic when associated with a net inward of ion of Na^+^ of sufficient magnitude to cause direct membrane depolarization. Transport is non-electrogenic when it activates intracellular cascades that in turn depolarizes the membrane for example via K^+^ conductance inhibition. The two receptor (R) families involved in nutrient sensing are: the large receptor family of seven transmembrane domains (**7TM)** named R1 and R2 in the figure and the smaller family of two transmembrane domains (**2TM**) named R3 in the schematic model. The main receptor family is composed of 7TM it could be observed as heterodimer, homodimer (R1) or monomer (R2). Nutrients binding to their receptors activate an intracellular cascade which induces membrane depolarization by activating (blue arrow) a Na^+^ influx or by inhibiting (red line) K^+^ conductance or hyperpolarization by the reverse events. Metabotropic (via intracellular cascades) activation and inhibition of ion channels induced by nutrients are represented by the blue and red dotted lines respectively.

In contrast to unicellular organisms, most eukaryotic cells are not directly exposed to changes in environmental nutrients. Nevertheless, nutrient homeostasis is essential for all living organisms to maintain constant fuel supply despite discontinuity in food intake. Nutrient scarcity and abundance exert a strong pressure on the selection of efficient mechanisms for nutrient sensing in mammalian cells including central neurons. However, the molecular nature of brain nutrient sensors has only recently started to be deciphered. The present review focuses on sensors that are present in olfactory areas. In particular, we present two major sensing mechanisms that involve either the family of solute carrier (SLC) transporters (called T in Figure 2) or receptors having seven or two transmembrane domains (called R in Figure 2).

In the first mechanism, the sensed molecule is transported intracellularly. Numerous transmembrane protein transporters belonging to the SLC superfamily have been associated with nutrient sensing that control feeding, energy expenditure, and counterregulation (Marty et al., 2007; Gonzalez et al., 2009; Routh, 2010; Broer, 2014). The SLC superfamily mediates passage of nutrients across the phospholipid bilayer via passive transport, in which the nutrient moves down its concentration gradient, or via active transport (or co-transport) that couples the movement of the nutrient to that of another molecule or ion crossing the membrane either in the same (symporter) or opposite direction (antiporter or exchanger). As a result, the membrane potential can be modulated directly when the sensed molecule is co-transported with ions (electrogenic transport) or indirectly when the sensed molecule activates an intracellular cascade which, in turn, modulates ion channel permeability (non-electrogenic transport).

In the second sensing mechanism, the sensed molecule binds to its transmembrane receptor and activates an intracellular cascade to depolarize the membrane through activation of Na^+^ and/or Ca^2+^ inflow or inhibition of K^+^ conductance (Lindemann, 2001; Chaudhari and Roper, 2010). In nutrient sensing, the most important transmembrane receptors belong to the seven transmembrane (7TM) G protein–coupled receptors (GPCRs) family and are activated by glucose, AAs, or FAs. These 7TM receptors are expressed in central nervous areas involved in energy homeostasis regulation (Wellendorph et al., 2010). The 7TM receptors exist across the phospholipid bilayer as homodimers, heterodimers, or monomers. It is noteworthy that a 2TM receptor called cluster of differentiation 36 (CD36), is often associated with FAs transporters in the hypothalamus (Doege and Stahl, 2006; Magnan et al., 2015).

## Glucose sensing

### Physiological role of glucose supply to the brain

Glucose is the primary metabolic substrate for the brain and a continuous supply of glucose is required for normal neuronal function (Mergenthaler et al., 2013). The brain accounts for 2% of the total body mass but requires 10 times more energy in the resting state compared to other energy consumption needs of the body (Mink et al., 1981; Molina and DiMaio, 2012). Glucose metabolism provides the fuel for physiological brain function through the generation of ATP that serves for the basic maintenance of cellular processes such as cytoskeletal dynamics, DNA repair, protein turnover, and growth. More specifically, during neuronal activation, the brain consumes a lot of energy in order to maintain the turnover of glutamate through metabolic neuron-astrocyte interactions (Magistretti and Allaman, 2015). Furthermore, 80% of total energy consumption fuels the Na^+^/K^+^ ATPase pump but <10% is used to recycle second messengers and neurotransmitters (Laughlin, 2001).

Glucose supply is critical for physiology, therefore a tight regulation between supply and demand is required. Several brain areas, such as the hypothalamus, brainstem, amygdala, septum, hippocampus, cortex, and OB contain glucose sensing neurons (Anand et al., 1964; Oomura et al., 1969; Ritter et al., 1981; Nakano et al., 1986; Shoji, 1992; Balfour et al., 2006; Tucker et al., 2013). These specialized neurons respond to local fluctuations in extracellular glucose levels, and modulate their mean firing rate accordingly. Glucose sensing neurons have been classified as “glucose-excited” (GE) or “glucose-inhibited” (GI) depending on whether they increase or decrease action potential frequency in response to extracellular glucose variations (McCrimmon, 2008; Gonzalez et al., 2009). GE and GI neurons integrate fluctuations in whole-body metabolic signals related to feeding behavior (Routh et al., 2007).

Several transporters, receptors, and ion channels are expressed in glucose sensing neurons of olfactory structures. Our laboratories and others have studied the role of the sodium-dependent glucose transporters (SGLTs), glucose transporters (GLUTs), potassium channels, and the insulin receptor (IR) in sensing glucose.

### Sensing role of glucose in olfactory structures: molecular hallmarks

#### Glucose transporters expressed in olfactory structures

##### Electrogenic solute carrier transporter (SGLT1)

**Figure 3:**
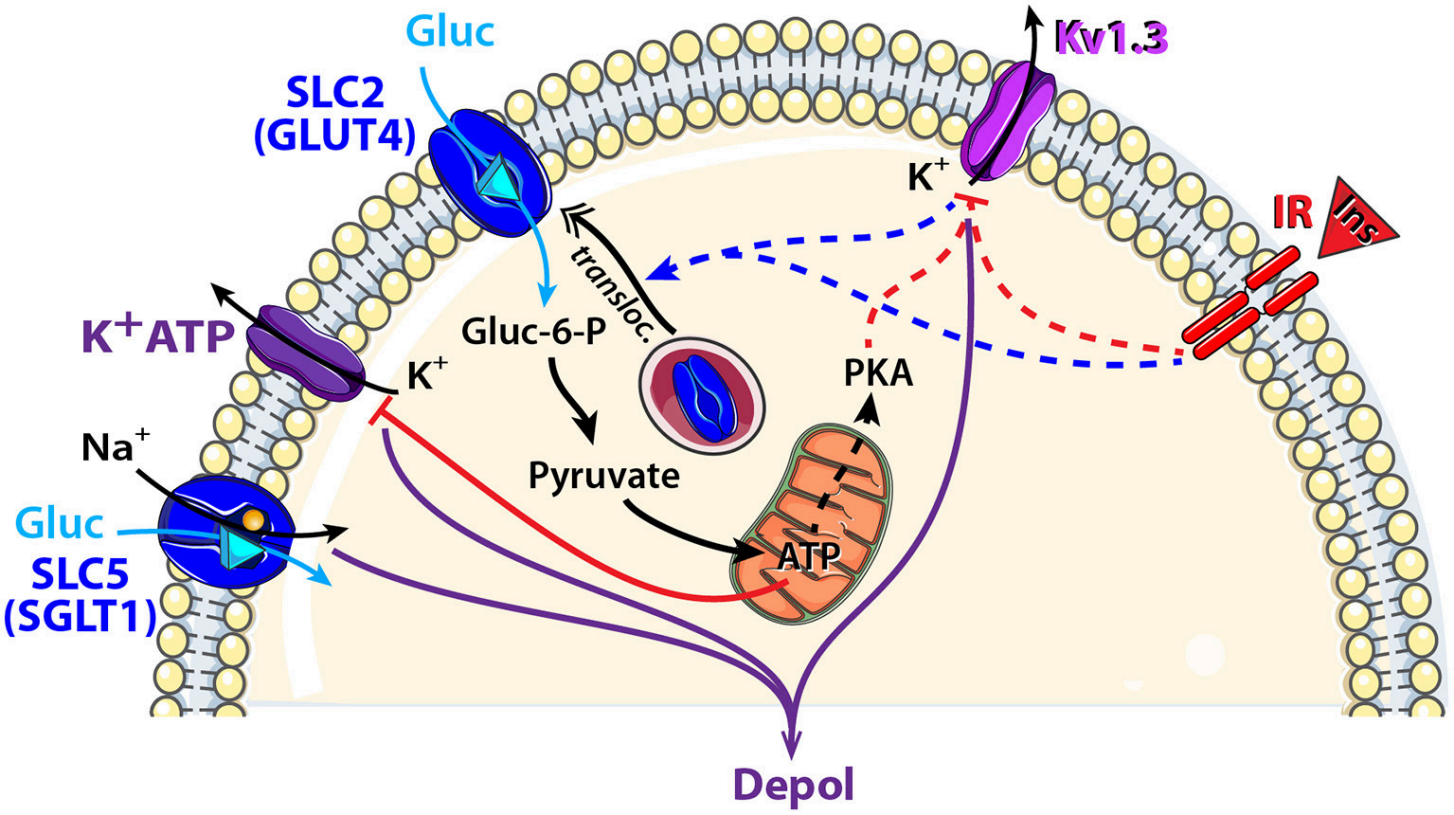
Schematic model showing glucose sensing signaling pathways that might modulate neuronal activity of central olfactory areas. Two types of glucose transporters and their associated downstream cellular processes are observed in central olfactory areas. SGLT1, located in the OB, is electrogenic and combines glucose (Gluc: blue triangle) translocation with an influx of Na^+^. GLUT4, located mainly in the OB and PC, is non-electrogenic and is associated with the insulin pathway. Indeed, insulin (Ins, red triangle) binding to its receptor (IR: insulin receptor) depolarizes MCs through Kv1.3 channel closure and induces GLUT4 translocation to the membrane. Glucose intake increases as well as the mitochondrial production of ATP and the cytosolic protein kinase A (PKA). Activation: blue arrow, inhibition: red line. Direct and indirect action of one molecule: full and dotted line respectively.

The family of sodium-dependent glucose transporters (SGLTs), also named SLC5, belongs to the SLC super family and uses a Na^+^ gradient to transport glucose against its concentration gradient into the cell. To date, six SGLTs isoforms have been identified (Wright and Turk, 2004). SGLT1 can modify its conformation to first release the two Na^+^ ions intracellularly while transporting glucose against its concentration gradient albeit in a symport orientation (Figure 3).

In the brain, SGLT1 has been found mainly in the hypothalamus, hippocampus, amygdala and OB (Kang et al., 2004; Yu et al., 2010; Aimé et al., 2014; Al Koborssy et al., 2014). In the OB, we found strong staining of SGLT1 in the inner part of the external plexiform layer (iEPL), in some mitral cells (MCs) and in some glomeruli (Al Koborssy et al., 2014). The iEPL is the site of reciprocal dendro-dendritic synapses between the secondary dendrites of MCs and the dendritic spines of inhibitory granule cells; this inhibitory interaction modulates odor information including olfactory discrimination (Yokoi et al., 1995; Lledo et al., 2005; Abraham et al., 2010). The availability of inhibitory control over MCs combined with the presence of rapidly activating SGLTs in the iEPL could explain the inhibitory response to glucose observed in the GI class of MCs (Tucker et al., 2013).

##### Non-electrogenic solute carrier transporter (GLUT4)

The Na^+^-independent GLUTs family (gene family *slc*2a) transports glucose across biological membranes. GLUTs comprise 14 family members and exhibit diverse substrate and tissue specificity resulting in distinct functional characteristics. GLUT1 exists as two isoforms in the brain and is exclusively expressed in endothelial cells and astrocytes. GLUT3 is localized to the neuropil and is largely absent from neuronal cell bodies (McCall et al., 1994; Gerhart et al., 1995) while GLUT4 exhibits a somato-dendritic labeling. The more discrete presence of GLUT4 compared with other GLUTs suggests that GLUT4 may be involved in highly specialized activities in the central nervous system. GLUT4 is consistently colocalized with IR and glucose transport through GLUT4 is the rate-limiting step in insulin-stimulated glucose uptake in the brain including olfactory areas (Alquier et al., 2006). Interestingly, 75% of GE neurons in the central nervous system coexpress GLUT4 and the IR mRNA (Kang et al., 2004).

The olfactory system has been found to express GLUT1 in the OE (Nunez-Parra et al., 2011), whereas GLUT1, GLUT3, and GLUT4 have been reported in the central olfactory areas (Brant et al., 1993; Leloup et al., 1996; El Messari et al., 1998, 2002; Vannucci et al., 1998; Dobrogowska and Vorbrodt, 1999; Choeiri et al., 2002; Al Koborssy et al., 2014). GLUT4 and IR are found to be localized in the main central olfactory areas such as the OB, PC, anterior olfactory nucleus (AON), and olfactory tubercle (OT) (Unger et al., 1989; Marks et al., 1990; El Messari et al., 1998; Schulingkamp et al., 2000; Alquier et al., 2006; Aimé et al., 2012, 2014). In a previous study, we have shown that GLUT4 is co-localized with IR in MCs and glomeruli of the OB. Interestingly, subcellular localization of GLUT4 is modulated by the feeding state. During the postprandial period when glucose levels in the blood are high, GLUT4 is found on the plasma membrane of dendritic processes. Following a fast however, it becomes internalized into the cytoplasm (Al Koborssy et al., 2014).

The dynamic expression of GLUT4 within MCs can be regulated by two complementary mechanisms (Figure 3). First, we observed that the feeding state-dependent modulation of GLUT4 subcellular localization in the OB correlates with the feeding state-dependent fluctuations of insulin levels in the OB as insulin was 2 fold higher in fed rats compared to fasted rats (Aimé et al., 2012). We infer that insulin levels increase in the OB during satiety to stimulate translocation of GLUT4 storage vesicles to the plasma membrane thereby increasing glucose uptake. Second, subcellular expression of GLUT4 can be regulated by the voltage-dependent potassium channel, Kv1.3 (Xu et al., 2004; Kovach et al., 2016). Blocking Kv1.3 conductance by applying a specific inhibitor (margatoxin) to cultured adipocytes or by co-transfecting GLUT4 and a non-conducting pore form of the channel in human embryonic kidney cells, increases plasma membrane expression of GLUT4 (Xu et al., 2004; Kovach et al., 2016). Gene-targeted deletion of Kv1.3 channel renders glucose-sensitive MCs non-responsive to glucose modulation in terms of action potential firing frequency (Tucker et al., 2013). Kv1.3 was further hypothesized to act as an insulin receptor substrate in MCs whereby IR activation phosphorylates the channel and suppresses its peak current (Fadool et al., 2000). It results that insulin-dependent or -independent blockade of Kv1.3 increases glucose translocation to the membrane.

While GLUT4 is highly expressed in MCs, and these neurons are clearly able to sense changes in glucose concentration either experimentally or evoked by nutritional state *in vivo*, the steps linking glucose entry to the change in firing pattern of MCs are yet unknown. We speculate that glucose sensing of MCs might use similar molecular means as reported for glucose sensing of the hypothalamus (Ashford et al., 1990; Spanswick et al., 1997; Ashcroft and Gribble, 1999; Song et al., 2001). In addition to K_ATP_, other transporters like the Na^+^/K^+^ ATPase pump (Oomura, 1983; Silver and Erecinska, 1998), and the cystic fibrosis transmembrane conductance regulator chloride channel (Hwang and Sheppard, 1999; Song et al., 2001) could elicit either depolarization or hyperpolarization of a neuron during extracellular glucose fluctuation.

Further studies are required to elucidate (i) if glucose transport across MCs recruits an electrogenic symport of Na^+^, (ii) if the metabolic product of glucose (ATP) acts on downstream ion channels similar to mechanisms observed in the hypothalamus or (iii) if byproducts of glucose metabolism could phosphorylate Kv1.3 through ATP, cAMP, or PKA (Lewis and Cahalan, 1995; Dalle et al., 2013).

### Metabolic dysfunction and glucose sensors in olfactory areas

A variety of functions have been suggested for central glucose sensing neurons. Glucose sensing neurons are involved (i) in maintaining local energy requirements for synaptic transmission and (ii) in regulating whole body energy and glucose homeostasis. Glucose not only serves as a metabolic substrate but also alters neuronal activity linked to metabolism. Therefore, it's suggested that correct functioning of glucose sensing neurons would be essential to prevent metabolic disorders such as obesity and Type 2 diabetes mellitus but also stroke and other neurodegenerative disorders where neuronal energy supply is disrupted (Routh et al., 2007).

Central olfactory areas such as the OB and PC, have an expensive energy budget in terms of glucose metabolism, which is high during odor stimulation and increases further during coding and processing of olfactory information (Nawroth et al., 2007; Gire et al., 2013; Litaudon et al., 2017). Given that, we previously established a link between feeding states and olfactory performance, and adding the dynamic changes in GLUT4 expression, insulin levels, and the numerous metabolic hormones present in the OB, we suggest that glucose sensing neurons are keys regulators of metabolic-dependent olfactory behavior.

In rodents, the concentration, expression, and activity of several molecules involved in glucose-sensing in olfactory areas are not only modified with feeding behavior but they are also altered by metabolic pathologies and their subsequent nutritional imbalance. In the OB, insulin concentration and GLUT4 expression are feeding-dependent but SGLT1 and IR expression are not (Aimé et al., 2012; Al Koborssy et al., 2014). In commonly used rodent models of obesity and type 2 diabetes, insulin concentration is elevated and SGLT1 is upregulated in the OB. Moreover, IR expression is down regulated but GLUT4 remained affected in both the OB and PC (Livingston et al., 1993; Vannucci et al., 1998; Aimé et al., 2014). Rodent models of obesity further display increased olfactory sensitivity and discrimination (Aimé et al., 2014; Chelminski et al., 2017).

We propose that dysregulation of glucose sensing markers could induce an increase in olfactory sensitivity which could lead to hyperphagia and metabolic disorders. These results suggest that dysfunctional glucose sensing neurons in the OB could alter olfactory pathways crucial to the regulation of food intake.

## Amino acid sensing

### Physiological role of amino acid supply to the brain

Amino acids (AAs) play a key physiological role as building blocks of proteins. Proteins not only play a structural role in the organism but they are involved in various metabolic processes, including enzymatic reactions. Among the 20 AAs that serve for protein synthesis, 10 are referred to as the essential AAs because they are acquired only from the diet and cannot be stored in the body. AA supply requires numerous membrane transporters and receptors that are tissue specific. Each carrier recognizes several AAs having structural similarities. In this manner, one AA is transported inside cells through multiple carriers with overlapping specificities (Taylor, 2014).

AAs are key regulators of metabolism (Wu, 2009). Homeostatic regulation of AA level is necessary to adapt AA concentration (essential and non-essential AAs) to physiological body requirements. In order to maintain an adequate AA supply, the hypothalamus senses AA notably through leucine detection that signals AA abundance and directly regulates food intake. Leucine intake activates the mammalian target of rapamycin complex 1 (mTORC1) and inhibits AMP-activated protein kinase (AMPK) in order to regulate protein translation and to reduce food intake (Cota et al., 2006; Ropelle et al., 2008). Indeed, central injection of leucine in the ventromedial hypothalamic nucleus has an anorectic effect through activation of a hypothalamic-brainstem circuit (Cota et al., 2006; Blouet et al., 2009; Haissaguerre et al., 2014). The nature of ingested AAs is also a very important parameter. Animals reject diet imbalanced in essential AAs, and forage for food with adequate AA content (Morrison et al., 2012; Anthony and Gietzen, 2013).

In the brain, AAs sensing could also implicate membrane receptors of GPCR family including the taste heterodimer receptor family (T1R1/T1R3) (Hoon et al., 1999; Li et al., 2002; Nelson et al., 2002) and CasR receptors (Conigrave et al., 2002).

The olfactory system plays a major role in AA sensing. The most studied mechanism uses SLC transporters but some receptors might also be implicated.

### Sensing role of amino acids in olfactory structures: molecular hallmarks

#### Amino acid transporters expressed in olfactory structures

**Figure 4:**
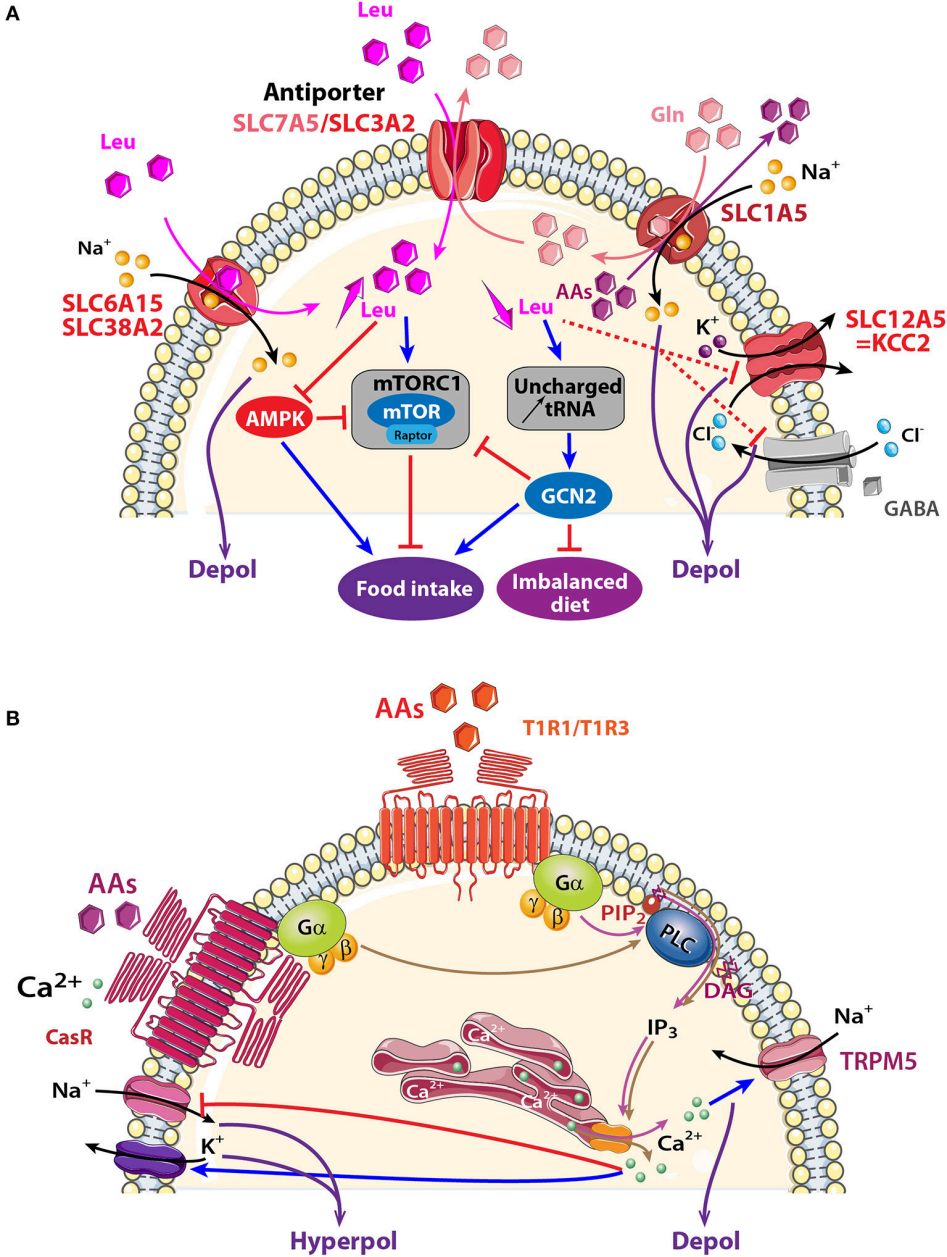
Schematic model showing AA sensing signaling pathways that might modulate neuronal activity of central olfactory areas. **(A)** Three electrogenic transporters (SLC6A15, SLC38A2, and SLC1A5) and one non-electrogenic antiporter SLC7A5/SLC3A2 are observed in the OB and the PC. AAs fluxes depend on physiological needs, on the importance of transported AAs (essential or non-essential), and on the cellular gradient of AAs. When leucine (Leu) and glutamine (Gln) are highly available, they are co-transported with sodium inside the cell through SLC6A15, SLC38A2 or SLC1A5. Intracellular Gln is in turn co-exchanged with Leu via the bidirectional antiporter SLC7A5/SLC3A2. The anterior PC (APC) detects essential AA deficiency that increases uncharged tRNA and activates the general amino acid control non-derepressible 2 (GCN2) pathway. The concomitant down regulation of GABA_*A*_ receptor and KCC2 transporter disinhibits the APC that send messages to nutritional brain areas in order to stop eating the imbalanced diet. Signaling proteins of the mammalian target of rapamycin complex1 (mTORC1) and AMP-activated protein kinase (AMPK) pathways are also present in olfactory areas, which suggests that these structures could also be implicated in detecting AA abundancy or scarcity and indirectly modulating food intake. **(B)** Two AA receptors are described: T1R1/T1R3, and CasR receptors. Both are G-protein-coupled receptors and AA binding activates heterotrimeric GTP-binding proteins composed of α-gustducin (Gα) and Gβγ subunits (brown and pink arrows). Gαpromotes phosphatidylinositol phosphate 2 (PIP_2_) activation of phospholipase C (PLC), leading to the production of inositol 1,4,5-trisphosphate (IP_3_) and diacylglycerol (D). IP_3_ opens ion channels on the endoplasmic reticulum, releasing Ca^2+^ into the cytosol of cells. Depending on the specific ion channels present on the membrane, a cell could be depolarized after melastatin-related transient receptor potential (TRPM5) channel opening or could be hyperpolarized after Na^+^ channel closure (red line) or Ca^2+^-dependent-K^+^ channel opening (blue arrow). AAs: hexagons; activation: blue arrow, inhibition: red line. Direct and indirect action of one molecule: full and dotted line respectively.

This chapter will focus attention on selected transporters that are known to be involved in metabolic regulation and are expressed in olfactory areas: the electrogenic transporters encoded by the *slc6a15, slc38a2*, and *slc1a5* genes and the non-electrogenic transporters encoded by *slc7a5* (Figure 4A).

##### Electrogenic solute carrier transporters (SLC6A15, SLC38A2, SLC1A5)

At least three electrogenic AA transporters are observed in olfactory areas (Figure 4A). They displace AAs together with Na^+^ and induce a subsequent depolarization.

Two of them, SLC6A15 and SLC38A2 transport small neutral AAs like leucine, isoleucine, and valine together with Na^+^ in a 1:1 stoichiometry (Yao et al., 2000; Mackenzie and Erickson, 2004; Broer et al., 2006; Hagglund et al., 2013). SLC6A15 is present in the OB, AON, and endopiriform and piriform cortices (Inoue et al., 1996; Masson et al., 1996; Drgonova et al., 2013; Hagglund et al., 2013; Allen Institute for Brain Science, 2015). SLC38A2 mRNA is three times higher in the OB than other brain areas like the hippocampus, hypothalamus, cortex, or PC (Sundberg et al., 2008; Allen Institute for Brain Science, 2015). SLC38A2 is associated with the general amino acid control non-derepressible 2 (GCN2) pathway (Blais et al., 2003; Palii et al., 2006; Gietzen and Aja, 2012; Taylor, 2014). This pathway is activated when essential AAs are deficient causing accumulation of uncharged tRNA (Zhang et al., 2002; Maurin et al., 2005; Gietzen and Aja, 2012). One or two hours after AA reduction, SLC38A2 synthesis is upregulated in order to increase AA uptake (Blais et al., 2003; Palii et al., 2006; Gietzen and Aja, 2012; Taylor, 2014). Deficiency in essential AAs affects the PC where it causes downregulation of GABA_*A*_ receptors and the K^+^/Cl^−^ co-transporter (KCC2), also known as SLC12A5 (Sharp et al., 2013). KCC2 is localized in GABAergic neurons in the OB and PC (Wang et al., 2005; Sharp et al., 2013). The PC is thus identified as the central structure that detects imbalanced diet lacking essential AAs. PC activation interrupts protein synthesis in 20 min and stops food intake in animals to promote foraging for a more appropriate diet (Leung et al., 1968; Koehnle et al., 2003; Gietzen and Aja, 2012; Morrison et al., 2012).

The third transporter, SLC1A5, is an antiport that exchanges one Na^+^ and glutamine against neutral AAs in a 1:1 stoichiometry (Kanai and Hediger, 2004; Nicklin et al., 2009; Pochini et al., 2014). SLC1A5 has long been considered an electroneutral transporter (Utsunomiya-Tate et al., 1996) but recently Scalise and collaborators suggested that more than one Na^+^ might be transported (Scalise et al., 2014). A wide distribution of the *slc1a5* is shown in MCs and the glomerular layer of the OB, and in the PC (Allen Institute for Brain Science, 2015). Glutamine and leucine intake through SLC1A5, together with SLC7A5/SLC3A2 (described in the next section), are proposed to be upstream steps of mTORC1 activation (Nicklin et al., 2009). The presence of these transporters in olfactory structures together with molecules involved in the mTORC1 pathway, such as raptor (Bar-Peled and Sabatini, 2014; Haissaguerre et al., 2014) makes it compelling to look for looking for AAs sensing through activation of the mTORC1 pathway in the olfactory system.

##### Non-electrogenic solute carrier transporter (SCL7A5/SLC3A2)

SLC7A5 is associated covalently with the glycoprotein SLC3A2. Both SLC7A5 and SLC3A2 are expressed in the OB, hippocampus, and hypothalamus (Kageyama et al., 2000; Allen Institute for Brain Science, 2015). SLC7A5/SLC3A2 is an AA exchanger that combines efflux of glutamine to influx of large neutral AAs like leucine with a 1:1 stoichiometry. Intracellular AA availability limits its transport rate given the low affinity of the intracellular domain of the transporter compared with its extracellular domain (Meier et al., 2002; Verrey, 2003). The net transport of AAs through SLC7A5/SLC3A2 is linked with electrogenic AA transporters like SLC1A5 that provides intracellular AAs for SLC7A5/SLC3A2 functioning. As a consequence, a reduced influx of glutamine through electrogenic transporters could limit leucine influx through SLC7A5 and consequently block the mTORC1 pathway (Verrey, 2003; Nicklin et al., 2009; Taylor, 2014).

### Amino acid receptors expressed in olfactory structures

#### Taste receptor family (T1R1/T1R3) expressed in olfactory structures

Taste buds of the tongue express the heterodimer receptor (T1R1/T1R3) belonging to a GPCR family that detects essential AAs (Hoon et al., 1999; Li et al., 2002; Nelson et al., 2002). *Tas1r1* and *Tas1r3* genes encoding for this receptor, and their associated G-proteins are found in a variety of central areas including the OB, hypothalamus and hippocampus, (Ren et al., 2009; Allen Institute for Brain Science, 2015; Voigt et al., 2015). Most members of the IP_3_ transduction pathway triggered by T1R1/T1R3 activation in the taste buds and the cation channel TRPM5 (Chaudhari et al., 2009; Chaudhari and Roper, 2010) are present in the OE, OB, and PC (Ross et al., 1989; Lin et al., 2007; Rolen et al., 2014; Allen Institute for Brain Science, 2015; Pyrski et al., 2017). In the future, studying the role played by T1R1/T1R3 in olfactory areas will be interesting in the context of AAs sensing (Figure 4B).

#### Calcium receptor family (CasR) expressed in olfactory structures

The localization and function of CasR in olfactory structures is species variant. In the OE of fish, CasR has the capacity to detect environmental Ca^2+^ and nutrients (Loretz, 2008). In rats, CasR transcript is expressed in the OB, AON and PC (Rogers et al., 1997; Ferry et al., 2000; Yano et al., 2004; Mudo et al., 2009). CasR is a multimodal receptor and it has been proposed to contribute to Ca^2+^ homeostasis and AA transport in neurons (Conigrave et al., 2002). When extracellular Ca^2+^ concentration reaches a threshold, CasR cooperatively binds to Ca^2+^ and to aromatic, aliphatic, or polar AAs (Conigrave et al., 2002; Conigrave and Hampson, 2006). Various intracellular pathways, including the downstream IP_3_ pathway, are activated to release internally stored Ca^2+^ (Hofer, 2005; Zhang et al., 2015). Excitability is reduced by opening Ca^2+^-dependent potassium channels and closing sodium channels (Han et al., 2015; Jones and Smith, 2016). The presence of CasR in olfactory structures together with components of IP_3_ pathway are good cues to investigate in the future if this transport allows olfactory structures to sense AAs.

### Metabolic dysfunction and amino acid sensors in olfactory areas

Taken together, the fact that olfactory areas express transporters, receptors and intracellular molecules implicated in the regulation of AA content, strongly suggests that the OB and PC could play an important role in AAs sensing.

When it comes to AA sensing via transporter activation, two mechanisms coexist: one involves the mTORC1/AMPK pathway that detects AA availability and the second one involves GCN2 that specifically alerts when one or more essential AAs are insufficiently ingested. The hypothalamus is proposed to be the center for mTORC1/AMPK signaling (Cota et al., 2006; Ropelle et al., 2008; Hagglund et al., 2013) while the anterior part of PC (APC) utilizes GCN2. Leung's and Gietzen's teams have collected convergent data showing that the APC is a sensor of AAs imbalanced diet. Briefly, deficiency in one essential AA induces rapid rejection of the imbalanced diet (Leung et al., 1968; Koehnle et al., 2003; Gietzen and Aja, 2012; Morrison et al., 2012). This aversion disappears after APC lesion (Leung and Rogers, 1971) and persists after hypothalamus or OB injury (Leung and Rogers, 1970; Leung et al., 1972), which identifies the APC as the sensor of an AA imbalanced diet. Moreover, local injection of the deficient AA in the APC reduces food aversion by maintaining consumption of the imbalanced diet (Beverly et al., 1990; Russell et al., 2003). Accumulation of uncharged tRNA caused by AAs deficiency activates the GCN2 pathway (Hao et al., 2005; Rudell et al., 2011) and disinhibits the APC mainly through downregulation of GABA_A_ receptor and KCC2, also known as SLC12A5 transporter (Sharp et al., 2013). KCC2 is localized in GABAergic neurons in the OB and PC (Wang et al., 2005; Sharp et al., 2013). Glutamatergic pyramidal neurons in the APC would then send messages to feeding circuits, including the hypothalamus, in order to stop food intake (Gietzen and Magrum, 2001). Noteworthy is that mTORC1 is not involved here because behavioral rejection of the improper diet remains in the presence of rapamycin (Hao et al., 2010) (Figure 4A).

The role played by the APC in sensing AA deficiency is thus clear. However, sensing AA abundance via other olfactory structures has not been explored yet. It would be interesting to explore the possible implication of OB and/or PC in detecting AA abundancy and scarcity through mTORC1/AMPK pathways and through AA receptor activation.

Another sensor of AAs, *Tas1R1*, seems to be dependent on the feeding state when expressed in the hypothalamus. *Tas1r1* levels increase following a 24-h food deprivation (Ren et al., 2009). *Tas1r1* is highly expressed in the hypothalamus of obese and hyperglycemic ob/ob mice. The similarities between the nutrient sensing properties of the hypothalamus and that of the OB (Figure 1) prompt further investigation of the role of T1R1 or the gene it encodes *Tas1r1*, in sensing AAs in olfactory structures.

## Fatty acid sensing

### Physiological role of fatty acid supply to the brain

The brain is roughly 50% fatty acids (FAs) by weight making it the organ with the second highest lipid content after that of adipose tissue (Watkins et al., 2001). Cerebral lipids are uptaken from the blood or synthesized locally (Rapoport et al., 2001; Smith and Nagura, 2001). Indeed, brain neurons express enzymes for both intracellular metabolism and *de novo* synthesis of FAs (Le Foll et al., 2009). In the human brain, the main source of polyunsaturated fatty acids (PUFAs) such as docosahexaenoic acid, eicosapentaenoic acid, and arachidonic acid, is dietary. Even though free FAs are not the primary metabolic fuel for neurons, they are key components of membranes and intracellular signaling pathways. PUFAs are of great importance in neurobiology because they are essential for neurogenesis, memory, learning, and play a key role in modulating ion channels and neurotransmitter receptors. In fact, an adequate lipid environment is vital for the normal functioning of neuronal membrane proteins such as ion channels, enzymes, ion pumps, and receptors. Long-term nutritional PUFA deficiency impairs brain functioning (Khan and He, 2017). FA sensing in neurons was first reported by Oomura et al. (1975). Since then, a growing body of evidence has established the importance of brain FA sensing in the regulation of food intake (Loftus et al., 2000; Lam et al., 2005; Levin et al., 2011). Specific areas of the central nervous system including the hypothalamus, brainstem, and hippocampus (Gao and Lane, 2003; Lam et al., 2005; Picard et al., 2014) have been shown to use FAs as cellular messengers to inform “FA-sensitive neurons” about the energy status of the body (Migrenne et al., 2011). Similar to glucose sensing and AAs sensing described previously, lipid sensing is involved in the control of feeding behavior (Obici and Rossetti, 2003; Cruciani-Guglielmacci et al., 2004). Hypothalamic lipid sensing mechanisms are disrupted during conditions of prolonged fasting (Yue and Lam, 2012). The molecular mechanisms involved in FA sensing by the brain are still a matter of debate.

The FA transporter proteins (FATP also called SLC27), is a protein family of six isoforms. SLC27A4 (FATP4) is the major FATP expressed in the brain (Fitscher et al., 1998). In hypothalamic neurons, FAs are transported inside cells through FATPs. FAs are then oxidized to generate ATP that can modulate the activity of a wide variety of ATP-dependent ion channels including K^+^ channels, and the Na^+^-K^+^ ATPase pump. The resulting change in neuronal firing rate suggests that FAs metabolism play a role in the regulation of energy balance (Migrenne et al., 2011; Picard et al., 2014).

In the brain, membrane receptors mediating FAs sensing consist of two GPCRs (GPR40 and GPR120) and CD36, often associated to fatty acid translocase (FAT) to make a translocator/receptor complex FAT/CD36. CD36 has been reported to be involved in FA sensing in taste buds (Fukuwatari et al., 1997; Laugerette et al., 2005) and in hypothalamic neurons (Le Foll et al., 2009). Hypothalamic CD36 expression induced by fasting or following high-fat diet, could modulate lipid signaling in the brain and participate in the regulation of energy homeostasis (Moulle et al., 2012, 2014). All together, these findings strongly suggest that lipid sensing by CD36 is responsible for basic physiological functions in relation to behavior and energy balance (Martin et al., 2011). In the hypothalamus, it has been postulated that binding of FAs to CD36 alters neuronal activity in a manner analogous to that utilized for fat perception by taste receptor cells (Le Foll et al., 2009). This causes phosphorylation of protein tyrosine kinases, leading to generation of IP_3_, recruitment of Ca^2+^ from the endoplasmic reticulum, followed by influx of calcium via opening of store-operated calcium channels, membrane depolarization via TRPM5 channel activation, and ultimately neurotransmitter release (El Yassimi et al., 2008).

In this review, only FA transporters (FATP/SLC27) and the FA receptors GPR40 and CD36 will be detailed. Intracellular proteins including long-chain fatty acyl-coenzyme A (CoA) synthetases and FA oxidative proteins are largely involved in neuronal FA sensing but are beyond the scope of this review (Picard et al., 2014).

### Sensing role of fatty acids in olfactory structures: molecular hallmarks

#### Fatty acid solute carrier transporters expressed in olfactory structures (SLC27)

**Figure 5:**
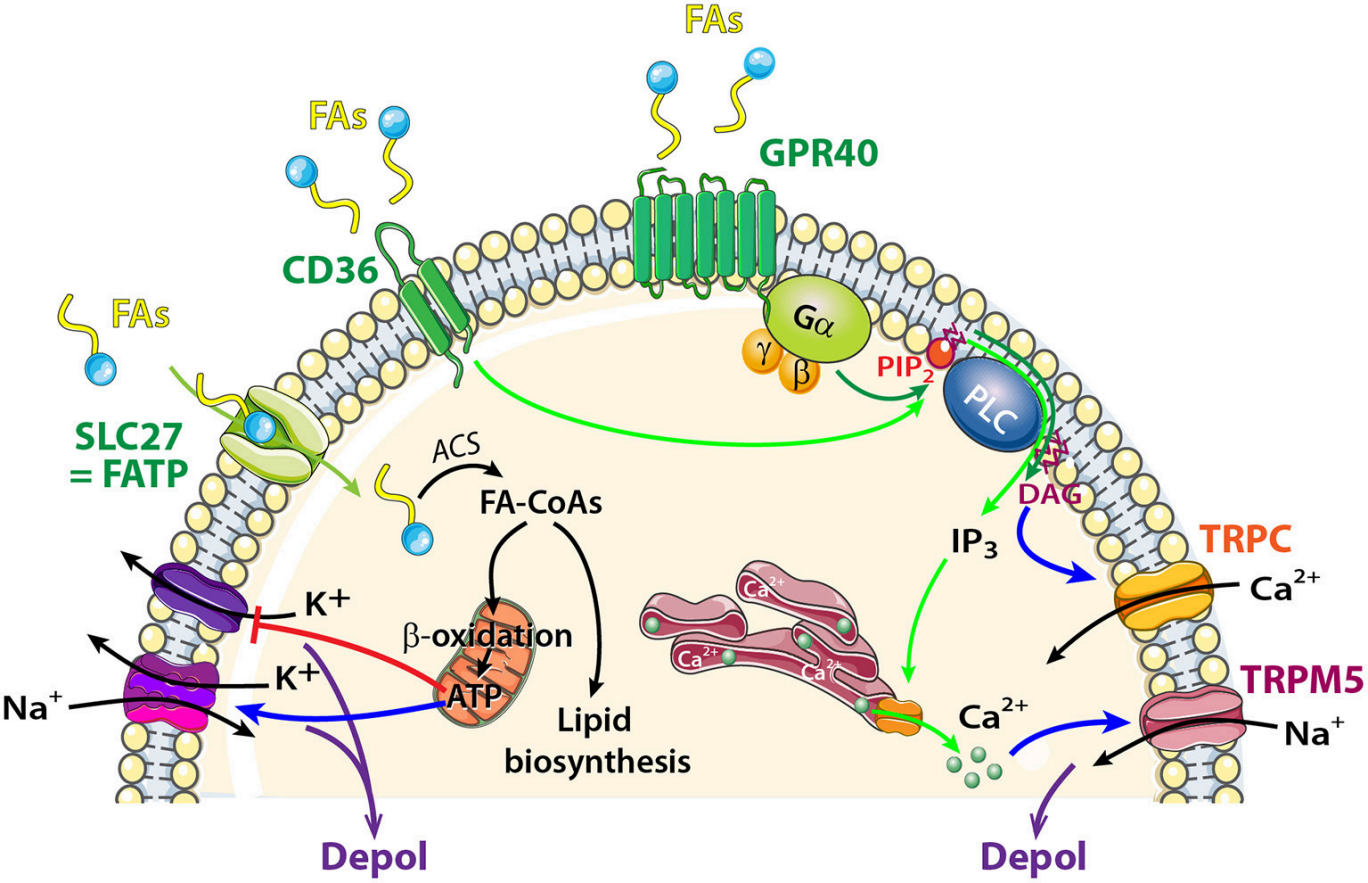
Schematic model showing FA sensing signaling pathways that might modulate neuronal activity of central olfactory areas. The transporter SLC27 induces influx of FAs, and acyl-CoA synthetase (ACS) to esterify FAs to fatty acyl-CoAs (FA-CoAs). Following mitochondrial β oxidation of FA-CoAs, production of ATP induces depolarization by acting on a wide variety of ATP dependent ion channels. FAs Receptors: Activation of CD36 by FA binding (light green arrows) causes phosphorylation of protein tyrosine kinases, leading to generation of inositol 1,4,5-trisphosphate (IP_3_) that induces Ca^2+^ release from the endoplasmic reticulum. [Ca^2+^]_*I*_increase depolarizes the membrane via TRPM5 channel. FAs receptors 7TM GPR40 receptor signaling (dark green arrows) acts through heterotrimeric G-proteins and produces IP3 and diacylglycerol (DAG). Phospholipase C (PLC) and DAG activate transient receptor potential cation channel subfamily C (TRPC).

According to the Allen Mouse Brain Atlas, SLC27A1 and SLC27A4 are expressed in the OB, AON, and PC. In the OB, SLC27A4 is mainly expressed in MCs (Allen Institute for Brain Science, 2015). While no previous study has investigated lipid sensing in central olfactory structures, many molecular cues seem to suggest that free FAs could be used as a messenger in these olfactory areas neurons to inform about the energy status of the whole body (Figure 5).

#### Fatty acid receptors expressed in olfactory structures

GPR40 (but not GPR120) is highly expressed in the OB (Nakamoto et al., 2012; Khan and He, 2017). Like all GPCRs, GPR40 is coupled to an intracellular heterotrimeric G protein (Gα) that activates the phospholipase C (PLC) located on the plasma membrane. PLC hydrolyzes phosphatidylinositol 4,5-bisphosphate (PIP_2_) into 2 s messengers: IP_3_ and diacyglycerol (DAG) (Figure 5). The generation of PLC facilitates transport of PKC from the cytosol to the plasma membrane. PLC, PKC, and DAG were described as activators of the TRP subfamily C (Khan and He, 2017). In the OB, MCs and external tufted cells extensively express TRPC3, C4, and C5 whereas neurons of the granule cell layer express TRPC1 and C4 only (Otsuka et al., 1998; Philipp et al., 1998; Dong et al., 2012). Studying modulation in MCs firing in response to fluctuations in extracellular FA concentration would be interesting in the context food intake and/or food choice.

In addition to GPRs, CD36 is a well described receptor for FAs. In the peripheral olfactory system, CD36 has been identified in insect and rodent OSNs (Benton et al., 2007; Lee et al., 2015). In recent studies, CD36 has been localized in the cilia, dendrites, and soma of a subset of OSNs in young rodents (Lee et al., 2015; Oberland et al., 2015). The CD36-positive OSNs respond in an age-dependent manner to oleic acid, a major milk component. This suggests that CD36 is involved in FA detection by the peripheral olfactory system during the suckling period (Oberland et al., 2015). CD36 was also found in central olfactory areas such as the glomerular layer of the OB (Oberland et al., 2015), PC and nucleus of the lateral olfactory tract (Glezer et al., 2009). The role of CD36 in these central olfactory areas has been raised whereby similar to taste buds, CD36 would sense FAs. TRPM5 channel is present in the OE, OB, and PC (Lin et al., 2007; Rolen et al., 2014; Allen Institute for Brain Science, 2015; Pyrski et al., 2017) and can serve as a downstream member of FA sensing where it is activated by an increase in Ca^2+^; the latter resulting from FA intake. CD36 activation would be investigated in the context of FAs sensing of olfactory areas.

### Metabolic dysfunction and lipid sensors in olfactory areas

In contrast to glucose and AAs sensing, only one study has explored the neuron lipid sensing in peripheral olfactory structures (Oberland et al., 2015). The fact that CD36, GPR40 and molecules involved in their intracellular pathways, are expressed in neurons of olfactory structures raises the question of their role(s) in lipid olfactory perception, central FA sensing, and regulation of energy balance. Indeed, lipid sensing is described as an important contributor to the regulation of energy balance (Magnan et al., 2015). In circumvallate taste buds, a decrease in CD36 expression induced by high-fat diet causes obesity and reduced sensitivity to fat taste, which in turn increased the intake of fatty foods as a compensatory response (Zhang et al., 2011). In the same way, reduction in hypothalamic CD36 expression induced redistribution of fat from visceral to subcutaneous deposits and markedly impaired insulin sensitivity (Le Foll et al., 2009, 2013, 2015). Growing evidence shows that dysregulation of brain FA sensing may contribute to energy imbalance and development of obesity, associated with type 2 diabetes or not (Yue and Lam, 2012; Picard et al., 2014). It will be interesting in future studies to investigate if olfactory dysfunction caused by altered energy balance (Thiebaud et al., 2014) could be linked to a change in expression of GPR40 and/or CD36.

## Conclusion

In order to regulate nutrient homeostasis, the body initiates multiple and redundant mechanisms in response to modulation in internal nutrient levels. In addition to the hypothalamic regulatory center, olfactory structures are proposed to detect both odors and nutrients. In this manner, the olfactory system contributes, through foraging and food, selection in maintaining metabolic homeostasis. In particular, mounting evidence indicates that the OB and the PC are involved in food intake, via regulation of choice of food with the appropriate nutrient content. This review presents a new approach to the problem of energy balance by suggesting that the nature of ingested nutrients could act on subpopulations of nutrient sensing neurons discreetly located in key brain areas including olfactory areas. In spite of numerous arguments described in this review (see Table 1), our understanding of the mechanisms implicated in nutrient sensing in olfactory areas is far from complete. The links between hormones involved in food intake regulation and that of nutrient sensing have to be deciphered. In the hypothalamus the mTORC1 is known to be a key component of the intracellular path integrating all these internal signals (i.e., nutrients and hormones) (Wullschleger et al., 2006; Wiczer and Thomas, 2010; Haissaguerre et al., 2014). We suggest that nutrient sensing in olfactory areas, could involve mTORC1 signaling. However, GCN2, and not mTORC1, is necessary for the detection of AA imbalance in the PC (Hao et al., 2010). The role of mTORC1 in detecting over consumption of nutrients in the PC, is a separate question to investigate. In addition to these and other unanswered questions, we still lack an integrative view of the presumably coordinated role played by olfactory areas and the hypothalamus regarding their metabolic homeostasis. Deciphering these aspects might offer new solutions in mitigating metabolic dysfunctions such as obesity and/or diabetes and provide new approaches to investigate physiological functions such as memory, and sleep that exhibit reciprocal relationships with homeostasis regulation and olfactory function (Barnes and Wilson, 2014).

**Table 1:** Overview of nutrient sensing molecular cues and their corresponding nutrients, present in olfactory structures.

**Nutrient**	**Nutrient sensing cues**	**Olfactory areas**	**References**
Glucose	GLUT3	OE, OB	Vannucci et al., 1998; Nunez-Parra et al., 2011
	GLUT4/IR	OB, AON, PC, OT	Leloup et al., 1996; El Messari et al., 1998, 2002; Vannucci et al., 1998; Choeiri et al., 2002; Aimé et al., 2014; Al Koborssy et al., 2014; Kovach et al., 2016
	SGLT1	OB	Aimé et al., 2014; Al Koborssy et al., 2014
	Kv1.3	OB	Tucker et al., 2010, 2013; Kovach et al., 2016
	mTORC1	OB, PC	Allen Institute for Brain Science, 2015
Amino acid	SLC7A5/SLC3A2	OB	Kageyama et al., 2000; Allen Institute for Brain Science, 2015
	SLC1A5	OB	Allen Institute for Brain Science, 2015
	SLC6A5	OB, AON, PC	Inoue et al., 1996; Masson et al., 1996; Drgonova et al., 2013; Hagglund et al., 2013
	SLC38A2	OB, PC	Sundberg et al., 2008; Allen Institute for Brain Science, 2015
	KCC2	PC	Wang et al., 2005; Sharp et al., 2013
	GCN2	PC	Maurin et al., 2005; Anthony and Gietzen, 2013
	mTORC1	OB, PC	Allen Institute for Brain Science, 2015
	T1R1	OB	Allen Institute for Brain Science, 2015; Voigt et al., 2015
	T1R3	OB	Allen Institute for Brain Science, 2015; Voigt et al., 2015
	TRPM5	OE, OB, PC	Lin et al., 2007; Rolen et al., 2014; Allen Institute for Brain Science, 2015; Pyrski et al., 2017
	GPCRs type CasR	OE	Loretz, 2008
		OB, AON, PC	Rogers et al., 1997; Ferry et al., 2000; Yano et al., 2004; Mudo et al., 2009
Fatty acid	SLC27A1, SLC27A4	OB, AON, PC	Allen Institute for Brain Science, 2015
	mTORC1	OB, PC	Allen Institute for Brain Science, 2015
	GPR40 (FFA1)	OB	Nakamoto et al., 2012; Khan and He, 2017
	CD36	OE, OB	Benton et al., 2007; Lee et al., 2015; Oberland et al., 2015
	TRPC	OB	Otsuka et al., 1998; Philipp et al., 1998; Dong et al., 2012
	TRPM5	OE, OB, PC	Lin et al., 2007; Rolen et al., 2014; Allen Institute for Brain Science, 2015; Pyrski et al., 2017

## Author contributions

AJ and BP were responsible for the conception and design of the review; DK, BP, and AJ drafted the review; All authors revised the manuscript critically for important intellectual content and approved the final version of the manuscript.

### Conflict of interest statement

The authors declare that the research was conducted in the absence of any commercial or financial relationships that could be construed as a potential conflict of interest.

## Abbreviations

CoA: Acyl-coenzyme A
AA: Amino acid
AON: Anterior olfactory nucleus
APC: Anterior piriform cortex
CD36: Cluster of Differentiation 36
DAG: Diacylglycerol
iEPL: Internal External plexiform layer
FA: Fatty acid
FAT: Fatty acid translocase
FATP: Fatty acid transport proteins
GCN2: General amino acid control non-derepressible 2
GE: Glucose-excited
GI: Glucose-inhibited
GLUT: Glucose transporter
GPCR or GPR: G-protein-coupled receptor
iEPL: Inner part of the external plexiform layer
IP_3_: Inositol 1,4,5-trisphosphate
IR: Insulin receptor
KCC2: K+/Cl- co-transporter
mTORC1: Mammalian target of rapamycin complex 1
MCs: Mitral cells
OB: Olfactory bulb
OE: Olfactory epithelium
OSN: Olfactory sensory neuron
OT: Olfactory tubercle
PIP_2_: Phosphatidylinositol 4,5-bisphosphate
PLC: Phospholipase C
PC: Piriform cortex
PUFA: Polyunsaturated fatty acid
7TM: Seven transmembrane domains
SGLT: Sodium-dependent glucose transporter
SLC: Solute carrier
TRPC: Transient receptor potential cation channel subfamily C
TRPM: Transient receptor potential cation channel subfamily M.
